# The Effects of Plant-Derived Oleanolic Acid on Selected Parameters of Glucose Homeostasis in a Diet-Induced Pre-Diabetic Rat Model

**DOI:** 10.3390/molecules23040794

**Published:** 2018-03-29

**Authors:** Mlindeli Gamede, Lindokuhle Mabuza, Phikelelani Ngubane, Andile Khathi

**Affiliations:** Schools of Laboratory Medicine and Medical Sciences, College of Health Sciences, University of KwaZulu-Natal, Durban 4004, South Africa; 213571877@stu.ukzn.ac.za (M.G.); 211509843@stu.ukzn.ac.za (L.M.); Ngubanep1@ukzn.ac.za (P.N.)

**Keywords:** oleanolic acid, high-fat high-carbohydrate diet, pre-diabetes, glucose homeostasis, insulin resistance

## Abstract

Prolonged exposure to high energy diets has been implicated in the development of pre-diabetes, a long-lasting condition that precedes type 2 diabetes mellitus (T2DM). A combination of pharmacological and dietary interventions is used to prevent the progression of pre-diabetes to T2DM. However, poor patient compliance leads to negligence of the dietary intervention and thus reduced drug efficiency. Oleanolic acid (OA) has been reported to possess anti-diabetic effects in type 1 diabetic rats. However, the effects of this compound on pre-diabetes have not yet been established. Consequently, this study sought to evaluate the effects OA on a diet-induced pre-diabetes rat model. Pre-diabetic male Sprague Dawley rats were treated with OA in both the presence and absence of dietary intervention for a period of 12 weeks. The administration of OA with and without dietary intervention resulted in significantly improved glucose homeostasis through reduced caloric intake, body weights, plasma ghrelin concentration and glycated haemoglobin by comparison to the pre-diabetic control. These results suggest that OA may be used to manage pre-diabetes as it was able to restore glucose homeostasis and prevented the progression to overt type 2 diabetes.

## 1. Introduction

The global prevalence of type two diabetes mellitus (T2DM) has increased rapidly and has been implicated in the increased prevalence of metabolic syndrome [1,2]. One of the predisposing risk factors that are associated with T2DM include an unhealthy lifestyle such as prolonged exposure to high energy diets [3]. However the progression from normoglycemia to the diabetic state encompasses a long-lasting intermediate period of moderate hyperglycaemia and increased levels of glycated haemoglobin known as pre-diabetic state [4]. These physiological derangements have been directly linked to the pathogenesis of T2DM [5]. Disruptions in the homeostasis of appetite-regulating hormones that lead to increased food intake have also been reported in pre-diabetes [6,7]. A recent study in our laboratory showed that animals fed a high-fat high-carbohydrate (HFHC) diet for 20 weeks develop pre-diabetes and various metabolic derangements including increased HbA1c, impaired glucose tolerance and elevated levels of plasma ghrelin [8]. Literature evidence indicates that the reported changes in these markers eventually lead to diabetes associated cardiovascular and renal complications [9]. Once diagnosed, pre-diabetes management relies on the combination of diet modification and administration of insulin sensitizers such as metformin [10,11]. However, over-reliance by patients on the pharmacological interventions has resulted in diet intervention being neglected and thus reducing the efficacy of the drugs [12,13]. Therefore, new compounds that will have the desired therapeutic effect even without the need for dietary intervention are required. OA and its derivatives have also been reported to upregulate the expression of GLUT4 which increases the glucose uptake in adipose and muscle cell lines [14,15] Previous studies in our laboratory have indicated that the plant-derived triterpene oleanolic acid (OA) possesses anti-hyperglycaemic properties and consequently suppress postprandial hyperglycaemia in a streptozotocin (STZ)-induced type 1 diabetic rat model [16,17]. In addition, OA has also been reported to work synergistically with insulin therapy in lowering blood glucose as well as ameliorating renal and hepatic dysfunction in STZ-induced type 1 diabetic rats [17,18]. However, the effects of OA on diet-induced pre-diabetes are yet to be investigated. Therefore, the current study sought to evaluate the effects of administering plant-derived OA with and without diet intervention on glucose handling as well as the metabolic hormones involved in glucose homeostasis in a diet-induced pre-diabetes animal model. 

## 2. Results

### 2.1. Caloric Intake

Food consumption of all experimental groups animals was determined every fourth week of the 12-week treatment period. The results showed that from the start of the treatment period (week 0), the PC group had a significantly higher caloric intake in comparison to NC (PC vs. NC) (*p* < 0.05). However, the administration of OA with and without dietary intervention resulted in a significant progressive decrease in caloric intake over the 12-week period by comparison to PC (Table 1).

### 2.2. Body Weights

**Figure 1:**
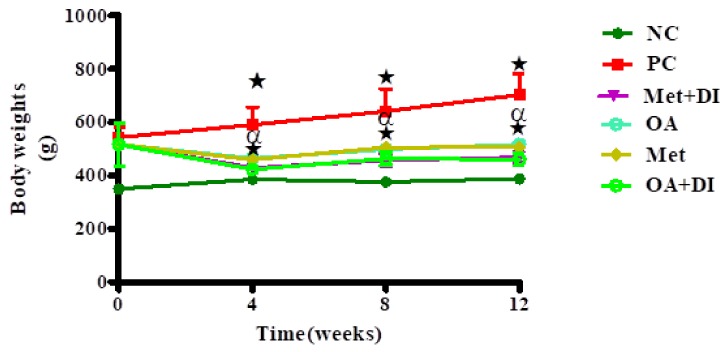
Effects of OA on body weights on rats that were treated OA with and without diet intervention during treatment period. Values are presented as standard error of mean ± SEM. ★ = *p* < 0.05 denotes comparison with NC; α = *p* < 0.05 denotes comparison with PC.

Body weights of all experimental groups were monitored every fourth week of the treatment period which lasted for 12 weeks. The results showed that from the start of the treatment period (week 0), the PC group had a significantly increased body weights in comparison to NC (PC vs. NC) (*p* < 0.05). However, the administration of OA with and without dietary intervention showed a significant decrease in body weights when compared to PC (*p* < 0.05) (Figure 1).

### 2.3. Oral Glucose Tolerance (OGTT)

**Figure 2:**
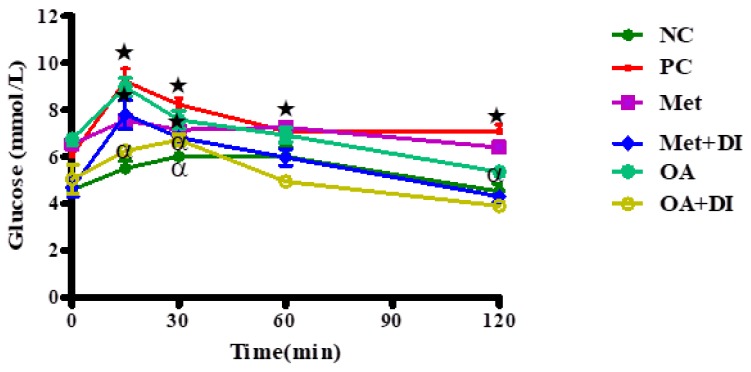
Effects of OA on OGTT of rats that were treated OA with and without diet intervention during treatment period. Values are presented as standard error of mean ± SEM. ★ = *p* < 0.05 denotes comparison with NC; α = *p* < 0.05 denotes comparison with PC.

The OGTT was conducted at the end of the treatment period (week 12) in all experimental groups. The results showed that at time 0, the PC had a significantly high blood glucose concentration when compared to NC and the same trend was observed for the duration of the experiment (*p* < 0.05). By the end of the experimental period, both OA-treated groups had a significantly lower blood glucose concentration when compared to PC. (*p* < 0.05) (Figure 2). 

### 2.4. HOMAR2-IR Index

At the end of treatment period (week 12), the HOMAR2-IR index of all animals was calculated using plasma glucose and insulin. The results showed that PC had a significantly higher HOMAR2-IR index when compared to NC (*p* < 0.05). Both OA-treated groups had a significantly lower HOMAR2-IR index in comparison to PC (*p* < 0.05) (Table 2). 

### 2.5. Glycated Haemoglobin Concentration (HbA1c)

**Figure 3:**
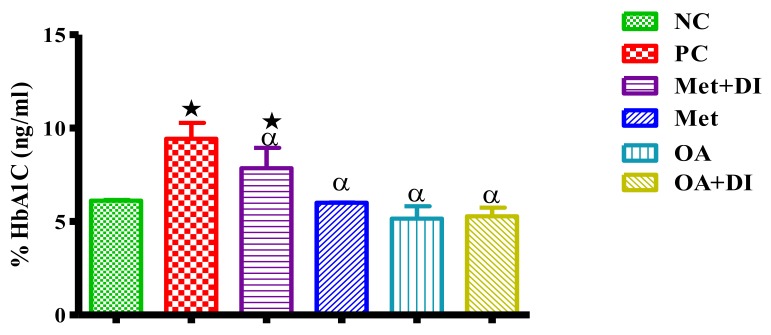
Effects of OA on the percentage of glycated haemoglobin concentrations of rats that continued with HFHC diet and those that changed diet or had diet intervention during treatment period. Values are presented as standard error of mean ± SEM. ★ = *p* < 0.05 denotes comparison with NC; α = *p* < 0.05 denotes comparison with PC.

All experimental groups were analyzed for HbA1c concentration at week 12. The results showed that whole blood HbA1c concentration of PC was significantly higher in comparison with NC (*p* < 0.05). The administration of OA with and without diet intervention resulted in a significant decrease in HbA1c concentration when compared to PC (*p* < 0.05) and these levels were comparable with the results of NC (Figure 3). 

### 2.6. Ghrelin Concentration

**Figure 4:**
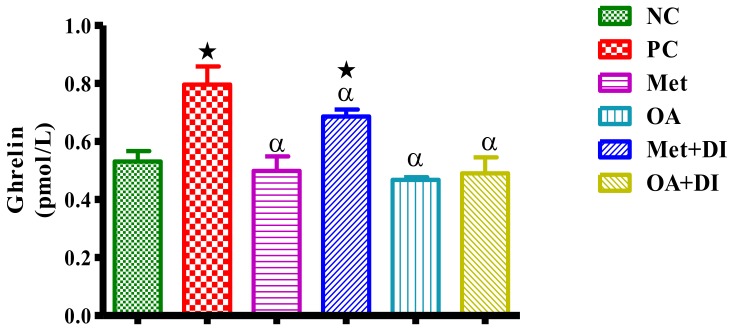
Effects of OA on plasma ghrelin concentrations of rats that continued with HFHC diet and those that changed diet or had diet intervention during treatment period. Values are presented as standard error of mean ± SEM. ★ = *p* < 0.05 denotes comparison with NC; α = *p* < 0.05 denotes comparison with PC.

Terminal plasma ghrelin concentrations of all experimental groups were measured at the end of treatment period. The results showed that PC had a significantly higher plasma ghrelin concentration in comparison to NC (*p* < 0.05). However, all OA-treated animals had a significantly lower ghrelin concentration when compared to PC (*p* < 0.05) (Figure 4).

### 2.7. Skeletal Muscle and Liver Glycogen Concentration

**Figure 5:**
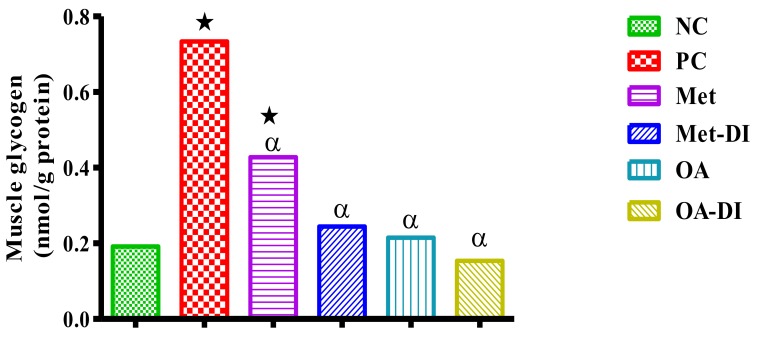
Effects of OA on muscle glycogen concentrations of rats that continued with HFHC diet and those that changed diet or had diet intervention during treatment period. Values are presented as mean. ★ = *p* < 0.05 denotes comparison with NC; α = *p* < 0.05 denotes comparison with PC.

**Figure 6:**
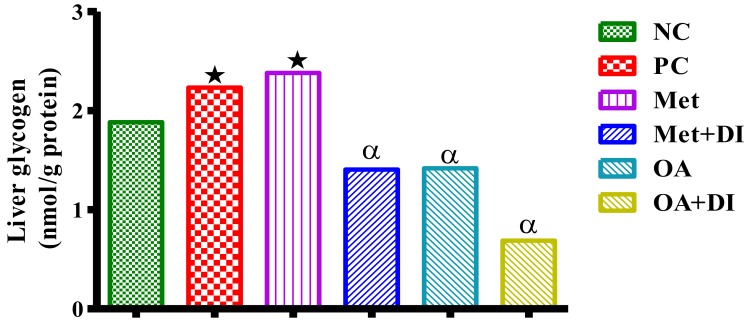
Effects of OA on liver glycogen concentrations of rats that continued with HFHC diet and those that changed diet or had diet intervention during treatment period. Values are presented as mean. ★ = *p* < 0.05 denotes comparison with NC; α = *p* < 0.05 denotes comparison with PC.

The glycogen concentration in the liver and skeletal muscle of all experimental groups was measured at the end of treatment period (week 12). The results showed that PC had significantly higher skeletal muscle and liver glycogen in comparison to NC (*p* < 0.05). However, treatment with OA resulted in a significant decrease in both skeletal muscle and liver glycogen concentrations when compared to PC (*p* < 0.05) Figure 5 and Figure 6.

## 3. Discussion 

The present study investigated the effects of *Syzygium aromaticum*-derived OA on glucose homeostasis in a diet-induced pre-diabetes rat model. A recent study from our laboratory reported that prolonged exposure of rats to a HFHC diet results in insulin resistance [19]. Previous studies have reported that that OA possesses synergistic pharmacological effects with insulin in STZ-induced type 1 diabetic rats as they were found to have anti-hyperglyaecemic, antiglycation, antioxidant and hepatoprotective properties [20]. To advance from these studies, we sought to investigate the possibility of reversing pre-diabetes primarily using oleanolic acid. Furthermore, dietary interventions are clinical recommendations that accompany the use of anti-diabetic drugs. This study therefore investigated the administration of OA in both the absence and presence of diet intervention. 

Under normal physiological conditions, ghrelin regulates energy metabolism through the regulation of food intake [21,22]. Ghrelin exerts orexigenic effects through activating hypothalamus receptors such as neuropeptide Y1 and Y5 while counteracting MC4R producing neurons [21]. In addition, this hormone also increases gamma aminobutyric acid (GABA) inhibitory postsynaptic currents and inhibitory synaptic contacts pre-opiomelano-cortin (POMC) neurons which counteract the effects of alpha-melanocyte stimulating hormone (αMSH) and further exacerbate orexigenic drive [23]. However, the administration of OA led to the reduction of plasma ghrelin concentration and the consequent reduction in caloric intake. Despite the unclear mechanism by which OA reduces circulating ghrelin concentration, we postulate that OA may restore ghrelin regulation through sensitizing the peripheral cells for insulin and suppress ghrelin secretion [20]. These findings of this study agree with the observations of an earlier study that showed that the administration of OA decreased ghrelin concentrations in STZ-induced type 1 diabetic rats [21]. Previous studies have shown that ghrelin also plays an important role in the regulation of glucose homeostasis through stimulating insulin release and sensitizing cells for insulin [22,24]. Ghrelin is also implicated in the inhibition of insulin stimulated glucose uptake during obesity [23]. Obeity is one of the predisposing factors for pre-diabetes as well as T2DM due to an increase in accumulation of ectopic fat deposition which is associated with insulin resistance [25]. However, several studies have shown that weight loss improves insulin sensitivity on obese individuals [26,27,28]. The regulation of caloric intake plays a crucial role in the management of metabolic disorders such as pre-diabetes and T2DM [29,30]. Furthermore, studies have shown that reducing caloric intake may play an important role in improving insulin sensitivity and subsequently the glucose homeostasis in pre-diabetic patients [31]. However, the modification of diets is often seen by patients as being of less significance and they tend to rely heavily on the medication [32,33]. In this study, OA mediated the reduction of caloric intake seen in this study in both the presence and absence of diet intervention resulting in decreased body weights and improvements in glucose tolerance. 

Impaired fasting glucose and glucose intolerance are some of the remarkable diagnostic features of pre-diabetes [34]. In this study, we observed that prolonged exposure to HFHC diet led to both impaired fasting glucose and impaired glucose tolerance. However, with the administration of OA we observed a decrease in fasting blood glucose concentrations and 2-h postprandial glucose concentrations to within the normal range. Previously, OA has been found to increase the expression of glucose transporters such as GLUT 4 in skeletal muscle of STZ-induced type 1 diabetic rats [35]. Another study reported that OA stimulates phosphoinositol-3-kinase which phosphorylates Akt and downregulates p-mTOR to improve insulin resistance [36]. Indeed, this study additionally showed that the administration of OA in both the presence and absence of diet intervention resulted in the reduction of plasma insulin concentrations. This was further evidenced by decreases in the HOMAR2-IR index which is used to quantify insulin resistance and beta-cell function [37].

Increased glycated haemoglobin (HbA1c) is also accepted as one of the diagnostic feature of pre-diabetes [38]. Increases in HbA1c develop because of sustained high plasma glucose concentrations which lead to glucose-mediated non-enzymatic glycation of haemoglobin [39]. In the present study, we observed that HFHC diet led to increased HbA1c indicating the moderate hyperglycaemia found in pre-diabetes [40]. However, the administration of OA decreased HbA1c and this may be due to OA sensitizing skeletal myocytes for insulin which led to translocation of GLUT 4 and subsequently the uptake of glucose by the cells [41]. Furthermore, these results suggest that OA started exerting these effects at the beginning of the study as HbA1c reflects average plasma glucose over the previous eight to 12 weeks [42]. Previous studies have reported that lowering HbA1c can be a sign that there is a sustained regulation of glucose homeostasis [42]. Taken together, the results of the study suggest that OA improves insulin sensitivity in diet-induced pre-diabetes even in the absence of diet intervention. 

## 4. Materials and Methods

### 4.1. Drugs and Chemicals 

All chemicals and reagents were sourced from the standard pharmaceutical suppliers and were of analytical grade.

### 4.2. Extraction Method and Administration

OA was extracted from *Syzygium aromaticum* ((Linnaeus) Merrill & Perry) (cloves) using an established protocol from [16]. Briefly: air-dried *S. aromaticum* flower buds (500 g) were milled and sequentially extracted twice at 24 h intervals at room temperature using 1 L dichloromethane (DCM), and ethyl acetate (720 mL) on each occasion. Subsequently, the extract was concentrated under reduced pressure at 55 ± 1 °C using a rotary evaporator to yield dichloromethane solubles (DCMS) and ethyl acetate solubles (EAS). The EAS containing mixtures of oleanolic/ursolic acid and methyl maslinate/methyl corosolate were purified by silica gel 60 column chromatography with hexane: ethyl acetate solvent systems of 7:3. This yielded OA which was further purified by recrystallization from chloroform-methanol (1:1, *v*/*v*). The structure of OA was confirmed by spectroscopic analysis using 1D and 2D, 1H and 13C nuclear magnetic resonance (NMR) spectroscopic experiments. The purity of the extracted OA used in the study was greater than 95%. 

### 4.3. Animals

Male Sprague-Dawley rats (130–160 g), bred and housed in the Biomedical Research Unit (BRU) of the University of KwaZulu-Natal were used in the study. All animal procedures and housing conditions was approved by the Animal Research Ethics Committee of the University of KwaZulu-Natal (ethics No.: AREC/035/016M). The animals were allowed access to food and fluids *ad libitum*. 

#### 4.3.1. Induction of Pre-Diabetes

Experimental pre-diabetes was induced in male Sprague-Dawley rats using a previously described protocol [19]. Briefly, the experimental animals were fed a high fat high carbohydrate (HFHC) diet supplemented with 15% fructose for 20 weeks while the control animals were exposed to standard chow for the equal number of weeks. After 20 weeks, the American Diabetes Federation criteria was used to diagnose pre-diabetes. The animals that were fed normal diet were also tested and were found to be normoglycemic and without pre-diabetes.

#### 4.3.2. Experimental Design

The study had two major groups which was the normal group and pre-diabetic group. Pre-diabetic group was further sub-divided into six groups each group having six rats (*n* = 6). The groups were categorized as follows: pre-diabetic control group (PC) which are the pre-diabetic animals continued with the experimental diet throughout the study period; metformin group (Met) which are the pre-diabetic animals that continued with the experimental diet but received metformin during treatment period; metformin and diet intervention group (Met + DI) which are the pre-diabetic animals that changed to a normal diet and received metformin during treatment period; oleanolic acid group (OA) which are the pre-diabetic animals that continued with experimental diet but received oleanolic acid during treatment period as well as OA and diet intervention group (OA + DI) which are the pre-diabetic animals that changed to a normal diet and received oleanolic acid during experimental period. The normal control group was the group of animals that were fed normal diet and diagnosed as without pre-diabetes. 

#### 4.3.3. Treatment of Pre-Diabetic Animals

The experimental period lasted for 12 weeks. The animals were treated every third day where the Met and Met + DI groups received (500 mg/kg) of metformin and the OA and OA + DI groups was given (80 mg/kg) of oleanolic acid. Parameters including food intake, body weights, fasting blood glucose were monitored at week 0, 4, 8 and 12. Glucose tolerance was also evaluated at week 12 with oral glucose tolerance test (OGTT) using OGTT protocol from previous studies in our laboratory [19].

#### 2.3.4. Blood Collection and Tissue Harvesting

After the experimental period, the animals were anaesthetized with Isofor (100 mg/kg) (Safeline Pharmaceuticals (Pty) Ltd., Roodeport, South Africa) via a gas anesthetic chamber (Biomedical Resource Unit, UKZN, Durban, South Africa) for 3 min. Blood was collected by cardiac puncture and then injected into individual pre-cooled heparinized containers. The blood was then centrifuged (Eppendorf centrifuge 5403, LGBW Germany) at 4 °C, 503× *g* for 15 min. Plasma was collected and stored at −80 °C in a Bio Ultra freezer (Snijers Scientific, Tilburg, NB, Netherlands) until ready for biochemical analysis. The harvested livers and skeletal muscle were rinsed with cold normal saline solution and snap frozen in liquid nitrogen before storage in a Bio Ultra freezer (Snijers Scientific) at −80 °C.

### 4.4. Biochemical Analysis

HbA1c and ghrelin concentrations were measured using their respective rat ELISA kits (Elabscience Biotechnology Co., Ltd., Houston, TX, USA) according to the manufacturer’s instructions. Plasma insulin concentration was measured using an ultra-sensitive rat insulin ELISA kit (Mercodia AB, Sylveniusgatan 8A, SE-754 50, Uppsala, Sweden) according to the manufacturer’s instructions. HOMAR-IR index was further calculated from insulin concentrations and fasting glucose. 

### 4.5. Glycogen Assay

Glycogen analysis was performed in muscle and liver tissues using a well-established laboratory protocol [16,35,43]. The harvested liver and muscle tissues were weighed and heated with potassium hydroxide (KOH) (30%, 2 mL) at 100 °C for 30 min. Then immediately disodium sulphite (Na_2_SO_4_) (10%, 0.194 mL) was added in to the mixture to stop the reaction. The mixture was then allowed to cool, and the glycogen precipitate was formed. The cooled mixture with precipitate was aspirated (200 µL) and mixed with ethanol (95%, 200 µL). The precipitated glycogen was pelleted, washed and resolubilized in H_2_O (1 mL). Thereafter, anthrone (0.5 g dissolve in 250 mL of sulphuric acid, 4 mL) was added and boiled for 10 min. After cooling the absorbance was read using the Spectrostar Nano spectrophotometer (BMG Labtech, Ortenburg, LGBW Germany) at 620 nm. The glycogen concentrations were calculated from the glycogen standard curve. The standard curve ranges from 200 to 1000 mg/L.

### 4.6. Statistical Analysis

All data was expressed as means ± S.E.M. Statistical comparisons were performed with GraphPadInStat Software (version 5.00, Graph Pad Software, Inc., San Diego, CA, USA) using one-way analysis of variance (ANOVA) followed by Steel-Dwass-Critchlow-Fligner multiple comparison test to simultaneously determine statistical differences between the means of two independent groups. A value of *p* < 0.05 was considered statistically significant.

## 5. Conclusions

The findings of this study confirm the previous findings that reported that OA has potential in managing diabetes. However, the findings of this study have revealed that OA can restore the regulation of glucose homeostasis in a diet-induced pre-diabetic rat model with and without the use of diet intervention. Therefore, this natural bioactive compound has shown potential in the prevention of the progression of pre-diabetes to T2DM. 

## Figures and Tables

**Table 1:** Effects of OA on food intake of rats that continued with HFHC diet during treatment and those that changed the diet. Values are presented as standard error of mean ± SEM and in percentage increase (↑ = increase and ↓ = decrease).

Caloric Intake (kcal/g)				
Experimental Groups	Week 0	Week 4	Week 8	Week 12
**NC**	109.18 ± 1.9 (**100%**)	125.042 ± 2.34 ↑ (**14.52%**)	165.04 ± 1.61 ↑ (**51.16%**)	178.40 ± 0.87 ↑ (**63.34%**)
**PC**	121.47 ± 1.01 * (**100%**)	120.90 ± 0.64 * ↓ (**0.47%**)	206.58 ± 0.84 * ↑ (**70.07%**)	230.01 ± 0.85 * ↑ (**89.36%**)
**Met**	118.09 ± 0.51 * (**100%**)	100.54 ± 0.98 * α↓ (**14.86%**)	99.51 ± 1.52 * α↓ (**15.73%**)	151.66 ± 0.69 * α↑ (**28.43%**)
**Met + DI**	115.02 ± 0.67 * (**100%**)	102.69 ± 1.17 * α↓ (**10.72%**)	120.51 ± 0.75 * α↑ (**4.77%**)	144.72 ± 1.64 * α↑ (**25.82%**)
**OA**	130.35 ± 0.03 * (**100%**)	103.94 ± 2.02 * α↓ (**20.26%**)	156.09 ± 1.63 * α↑ (**19.75%**)	194.26 ± 1.85 * α↑ (**49.03%**)
**OA + DI**	117.58 ± 0.51 * (**100%**)	118.04 ± 0.85 *α ↑ (**0.40%**)	147.59 ± 2.74 *α ↑ (**25.52%**)	168.82 ± 2.22α ↑ (**43.58%**)

* *p* < 0.05 denotes comparison with NC; α *p* < 0.05 denotes comparison with PC.

**Table 2:** Effects of OA with and without diet intervention on HOMAR index after the treatment period. Values are presented as standard error of mean ± SEM.

HOMAR2-IR Index			
Experimental Group	Plasma Glucose (mmol/L)	Plasma Insulin (mU/L)	HOMAR2-IR Values
**NC**	4.60 ± 0.09	61.96 ± 0.90	12.670 ± 0.61
**PC**	5.87 ± 0.32 *	491.64 ± 3.45 *	128.26 ± 2.98 *
**Met**	6.55 ± 0.81 *α	169.16 ± 3.12 *α	15.93 ± 1.02 *α
**Met + DI**	4.52 ± 0.90α	142.94 ± *α	49.25 ± 1.15 *α
**OA**	6.77 ± 1.59 *α	200.58 ± 2.85 *α	60.35 ± 2.05 *α
**OA + DI**	4.47 ± 0.12α	68.59 ± 2.01α	13.63 ± 0.95α

* *p* < 0.05 denotes comparison with NC; α *p* < 0.05 denotes comparison with PC.
